# Application of quantum dot nanoparticles for potential non-invasive bio-imaging of mammalian spermatozoa

**DOI:** 10.1186/1477-3155-10-45

**Published:** 2012-12-14

**Authors:** Jean M Feugang, Ramey C Youngblood, Jonathan M Greene, Abed S Fahad, William A Monroe, Scott T Willard, Peter L Ryan

**Affiliations:** 1 Facility for Cellular Imaging and Organismal Imaging, Mississippi State University, Mississippi State, MS, USA; 2 Department of Animal and Dairy Sciences, Mississippi State University, Mississippi State, MS, USA; 3 Department of Pathology and Population Medicine, College of Veterinary and Medicine, Mississippi State, USA; 4 Institute for Imaging and Analytical Technologies, Mississippi State University, Mississippi State, MS, USA; 5 Department of Biochemistry and Molecular Biology, Entomology and Soil Sciences, Mississippi State University, Mississippi State, MS, USA

**Keywords:** Spermatozoa, Fertilization, Quantum dot, Nanoparticles, Biophotonic imaging, Bioluminescence imaging

## Abstract

**Background:**

Various obstacles are encountered by mammalian spermatozoa during their journey through the female genital tract, and only few or none will reach the site of fertilization. Currently, there are limited technical approaches for non-invasive investigation of spermatozoa migration after insemination. As the knowledge surrounding sperm behavior throughout the female genital tract still remains elusive, the recent development of self-illuminating quantum dot nanoparticles may present a potential means for real-time *in vitro* and *in vivo* monitoring of spermatozoa.

**Results:**

Here, we show the ability of boar spermatozoa to harmlessly interact and incorporate bioluminescent resonance energy transfer-conjugated quantum dot (BRET-QD) nanoparticles. The confocal microscope revealed *in situ* fluorescence of BRET-QD in the entire spermatozoon, while the ultra-structural analysis using the transmission electron microscope indicated BRET-QD localization on the sperm plasma membrane and intracellular compartment. In controlled-*in vitro* assays, bioluminescent imaging demonstrated that spermatozoa incubated with BRET-QD and luciferase substrate (coelenterazine) emit light (photons/sec) above the background, which confirmed the *in situ* fluorescence imaging. Most importantly, sperm motility, viability, and fertilizing potential were not affected by the BRET-QD incorporation when used at an appropriated ratio.

**Conclusions:**

Our results demonstrate that pig spermatozoa can incorporate BRET-QD nanoparticles without affecting their motility and capacity to interact with the oocyte when used at an appropriated balance. We anticipate that our study will enable in-depth exploration of the male components of *in vivo* migration, fertilization, and embryonic development at the molecular level using this novel approach.

## Background

Mammalian spermatozoa are tiny and highly specialized cells shaped to enable migration through the female genital tract, interact with oocytes, and deliver the paternal materials to the oocyte. However, various obstacles associated with spermatozoa themselves or encountered within the female genital tract may lead to few or no spermatozoa reaching the site of fertilization [1-3]. This situation inevitably affects the pregnancy outcome and there is a crucial need to better understand the sperm behavior within the female genital tract (i.e., migration and interactions with its surrounding environment), as well as the molecular and cellular events that precede fertilization *in vivo*.

At present, the conventional experimental approaches of studying mammalian spermatozoa are limited by researchers’ inability to accurately and non-invasively investigate sperm quality and viability before and after insemination [4-7], as the normal sperm progression within the female genital tract remains unclear. The development of new techniques that enable non-invasive monitoring of sperm movement after artificial insemination has the potential to enhance breeding efficiencies, which could be achieved by either selecting sires with spermatozoa more apt to overcome utero-oviductal hindrances and encounter oocytes *in vivo* or dams that are more likely to facilitate the migration and interaction of both gametes. A recent study towards this goal has successfully imaged living ram spermatozoa in different settings (*in vitro*, *ex vivo*, and *in vivo*) using organic fluorochromes, which have limitations in terms of brightness and photo-stability for deep-tissue imaging [8,9].

As an alternative to traditional organic fluorescent dyes, such as green fluorescence protein, the recent progress in the nanotechnology field has led to the production of biocompatible quantum dot (QD) nanoparticles that are highly photo-stable and brighter [10]. These nanoparticles can be produced in various sizes to emit a vast spectra of wavelengths upon a single excitation [11] and, therefore, permit their utilization in various areas of biomedicine for targeted and non-targeted *in vitro* and *in vivo* imaging [10,12-14]. Most interestingly, the ability of QD to fluoresce in the near infra-red spectrum and to be linked to a variety of substances (i.e., peptides, nucleic acids, and luciferase) creates more opportunities for these nanoparticles [11,15,16]. At present, the nanotechnology has not been applied in the field of reproductive biology while it could be useful for molecular imaging. We believe this technology can provide invaluable insight into biological and cellular processes associated with gamete behavior and interactions, and early embryo development.

In this study, we explored the potential use of QD nanoparticles as a flexible tool to apply for non-invasive investigation of mammalian spermatozoa. Quantum dots emitting at 655 nm wavelength and conjugated with *Renilla* luciferase and nona-arginine R9 internalization peptide (BRET-QD; [17]) were used to label boar spermatozoa, followed by the assessment of their impact on sperm motility, viability, and fertilizing potential.

## Results and discussion

Here, we investigated the ability of mammalian spermatozoa to harmlessly incorporate CdSe/ZnS QD nanoparticles conjugated to the nona-Arginine R9 peptide that facilitates its cellular internalization. For bio-imaging purpose, QD were linked to the *Renilla* luciferase enzyme which in the presence of its substrate, coelenterazine, creates a self-illuminating QD-Bioluminescent Resonance Energy Transfer complex (BRET-QD) emitting both light and fluorescence that are captured with appropriate equipment.

### Evaluation of BRET-QD internalization in spermatozoa

**Figure 1:**
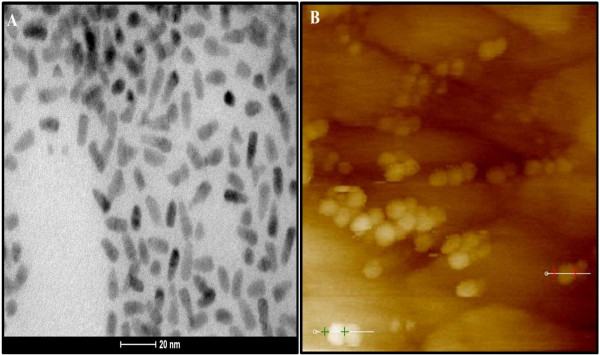
**Assessment of the BRET-QD size.** BRET-QD analyzed with Transmission Electron Microscope (**A**) and Atomic Force Microscope (**B**). Scale bars = 20 nm.

**Figure 2:**
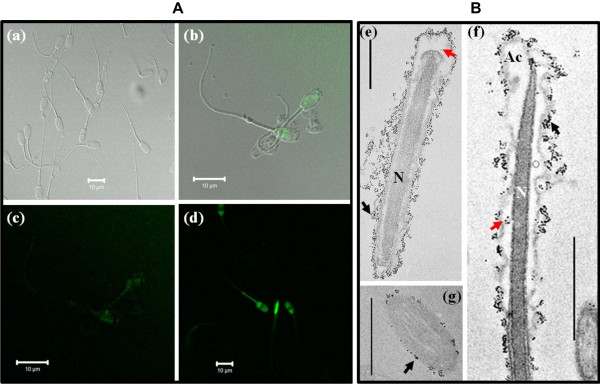
**Localization of BRET-QD in boar spermatozoa.** (**A**) Detection of QD fluorescence using Confocal Microscopy. Overlays of bright field and fluorescence lights corresponding to spermatozoa incubated with 0 and 1 nM BRET-QD are shown in (**a**) and (**b**), respectively. Fluorescence detection in spermatozoa incubated with 1 or 5 nM BRET-QD is shown in (**c**) and (**d**), respectively. (**B**) Localization of QD using Transmission Electron Microscopy (TEM). Micrographs (**e**) and (**f**) respectively show transversal and longitudinal cross sections of the head. A transversal cross section of the tail is shown in (**g**). Red and black arrows respectively indicate QD within the cytoplasm and the surface plasma membrane. Acrosome and nucleus areas are indicated as Ac and N, respectively. Scale bars = 10 μm in (**a**), (**b**), (**c**) and (**d**), or 0.5 μm in (**e**), (**f**) and (**g**).

We first measured the size of the QD’s core-shell (CdSe/ZnS) that was found around 5 to 7 nm using transmission electron microscopy (TEM; Figure 1A), while the entire BRET-QD was approximately 20 nm to 25 nm using atomic force microscopy (AFM; Figure 1B). These observations were in agreement with our expectations and previous reports [15,18,19]. Therefore, the BRET-QD was used as a biological probe to label and track boar spermatozoa *in vitro*. Confocal laser scanning microscopy revealed a dose-dependent fluorescence of BRET-QD in spermatozoa, with a higher signal emission observed in those exposed to 5 nM (Figure 2d) compared to their counterparts labeled with 1 nM (Figure 2b-c) or 0 nM BRET-QD (Figure 2a). Spermatozoa labeled with 5 nM BRET-QD displayed a stronger and well-distributed fluorescence throughout the entire spermatozoon, while those exposed to 1nM possessed a fluorescence signal mostly located in the head and mid-piece regions. There was no fluorescence detected in samples incubated without BRET-QD (Figure 2a). Furthermore, the TEM analysis also confirmed the presence of BRET-QD in labeled-spermatozoa (Figure 2e-g). In this later technique, the sperm preparation did not include osmium tetroxide post-fixation and uranyl acetate and lead citrate staining [20], which allowed a better background contrast with the BRET-QD signal. Surprisingly, the majority of QD nanoparticles appeared on the sperm plasma membrane, while fewer were found in the head’s cytoplasm. This distribution of BRET-QD was not expected given the reported efficiency of R9 peptide to cargo molecules into somatic cells in a short period, of approximately 4 min [21,22]. Because the cellular internalization of R9 peptide is energy-independent and does not require receptors for plasma membrane penetration, we can speculate that this peptide may be less efficient in spermatozoa due to the special composition of their plasma membrane [23,24], or that the incubation time applied in our study (30 min) was not sufficient for a greater enrichment of spermatozoa with the nanoparticles. For further studies, additional enrichment could be reached by increasing the (BRET-QD/Spermatozoa) co-incubation time or by replacing the cell penetrating peptide carrier. Indeed, the HR9 (histidine-rich) peptide has been shown to be more efficient than the R9 (arginine-rich) for intracellular delivery of nanoparticles [25].

### Bioluminescence imaging of spermatozoa exposed to BRET-QD

**Figure 3:**
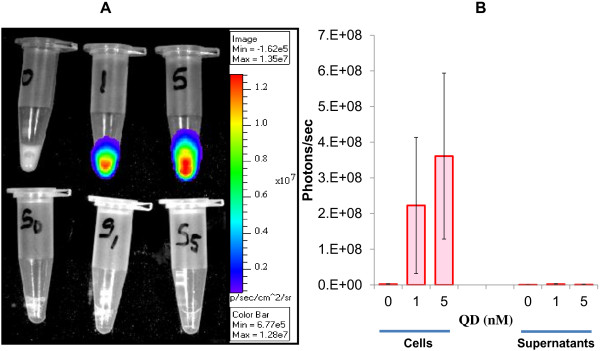
**Detection of BRET-QD bioluminescence in spermatozoa.** (**A**) Representative bioluminescence signals (photons/sec) of 0, 1 or 5 nM BRET-QD in spermatozoa (upper panel) and corresponding washing/supernatant media (bottom panel). (**B**) Bioluminescence signal quantification (mean ± s.d.) of 4 independent replicates.

The BRET conjugate offers the possibility for approximate and non-invasive quantification of cell population size. Here, we used the IVIS bioluminescence system to image the successful interaction between spermatozoa and BRET-QD (Figure 3). In this *in vitro*-controlled experiment using fixed amounts of sperm cells (10^8^), the bioluminescent signal (photons/sec) was detected only in spermatozoa exposed to both the BRET-QD (1 nM and 5 nM) and the luciferase substrate (coelenterazine) (Figure 3A). There was no light signal detected in the control group (0 nM), and labeled-spermatozoa exhibited light intensities that appeared in a dose-dependent manner, although we could not find a statistical difference between both exposed-groups (Figure 3B). Interestingly, the intensities of light emitted in corresponding supernatants were detected at a background level, similar to the signal in the control group. This later observation indicated that the bioluminescence signal detected in labeled-spermatozoa derived exclusively from incorporated BRET-QD. In our knowledge, this is a first report of BRET-QD labeled-spermatozoa.

**Figure 4:**
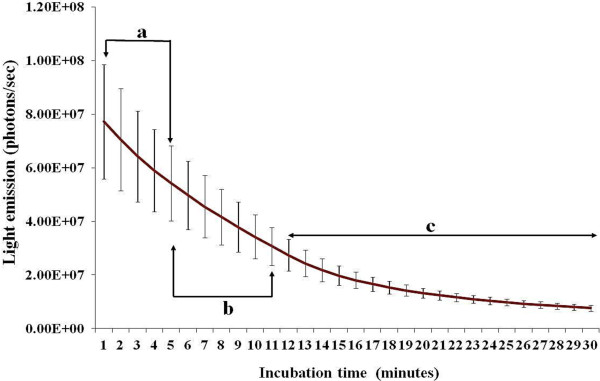
**Light decay after addition of coelenterazine.** Time-points under similar letters (**a,b,c**) do not significantly differ (ANOVA-repeated measurements). Data are means (± s.e.m.) of 4 independent replicates.

The lack of a significant difference between labeled-groups (1 nM and 5 nM) prompted us to evaluate the rate of light decay after addition of coelenterazine. We found a significant decrease of BRET signal overtime that reached approximately 50% within 10 min after the addition of coelenterazine (Figure 4). This rapid falloff of BRET intensity was also reported in previous studies [26,27]. In our conditions, imaging was usually performed around 5 min of coelenterazine supplementation and, although high levels of signal were still detectable, approximately 30% of the initial signal was already lost at this time point; and variations in incubation periods among replicates may explain the lack of statistical differences between labeled-spermatozoa groups (1 nM and 5 nM) as shown in Figure 3B.

Altogether, our data indicate that large amounts of BRET-QD can interact with living mammalian spermatozoa, which is of great interest for *in vivo* imaging. Nonetheless, the ability of these nano-sized particles to enter cells may cause unexpected toxicities which have already been reported in somatic cells [28,29].

### Assessment of BRET-QD internalization on sperm viability and fertilizing potential

As a first step to assess the potential toxicity of BRET-QD, we evaluated the motility, viability and fertilizing potential of spermatozoa after incubation (30 min) with BRET-QD.

As for the motility study, spermatozoa were incubated at various concentrations (0.1x, 0.5x, 1x, and 2x 10^8^ sperm/ml) with a fixed concentration of BRET-QD (1 nM). The incorporation of BRET-QD was confirmed by bioluminescence imaging (as shown above). Results in Table 1 indicate the significant reduction of the proportions of motile and rapid spermatozoa in 0.1x and 0.5x 10^8^/ml groups (P < 0.05), while the corresponding velocity parameters (VAP, VCL, and VSL) tended to decrease (P < 0.1). Although we could not demonstrate it, we speculated that the significant falls in both parameters were due to an overload of spermatozoa with BRET-QD rather than a potential toxicity. We did not perform any biochemical assays (i.e., apoptosis) to confirm our speculation, but previous studies conducted in mouse oocytes and somatic cells already reported the non-toxicity effect of QD used at concentrations less than 200 nM [22,30]. These authors showed that the coating of CdSe-core QD with the ZnS shell restored the detrimental effects of 500 nM CdSe-core QD on the oocyte developmental competence [30]. Furthermore, results in Table 1 also indicates that the motility and velocity parameters of spermatozoa incubated at 1x 10^8^ and 2x 10^8^/ml with BRET-QD (1 nM) were comparable to those obtained in the control group (P > 0.05). The control group corresponded to the pool of various concentrations of non-labeled-spermatozoa which consistently had comparable data in five consecutive replicates. Overall, these data suggest that a balanced equilibrium between BRET-QD and sperm concentration is crucial to maintain both the motility and the velocity of spermatozoa.

**Table 1:** Effect of BRET-QD and sperm ratio on sperm motility

**Groups**	**N**	**Motility**^**1**^**(%)**	**Rapid**^**2**^**(%)**	**VAP (μm/sec)**	**VCL (μm/sec)**	**VSL (μm/sec)**
Control^*^	5	87 ± 6^a^	74 ± 5	94 ± 11	202 ± 23^a^	56 ± 9
0.1 x 10^8^	3	36 ± 6^b^	23 ± 13	40 ± 5	86 ± 15^b^	21 ± 5
0.5 x 10^8^	4	63 ± 17^ab^	46 ± 15	65 ± 19	143 ± 36^ab^	39 ± 12
1 x 10^8^	4	81 ± 8^a^	68 ± 11	90 ± 9	194 ± 17^a^	51 ± 7
2 x 10^8^	3	89 ± 5^a^	63 ± 22	94 ± 17	198 ± 36^a^	52 ± 8
*P values (ANOVA 2)*		*0.017*	*0.094*	*0.069*	*0.053*	*0.116*

^*^Spermatozoa incubated without BRET-QD (1 nM). N = number of independent replicates; ^1^Total motility of spermatozoa; ^2^Proportion of sperm moving at a speed ≥ 30 μm/sec. Velocity data correspond to the average path (VAP), curvilinear (VCL), and straight-line (VSL). Data are mean ± s.e.m. and superscripts (^a,b^) indicate significant differences within the same column.

Even though these parameters are maintained, it is still reasonable to believe that the BRET-QD may perturb the membrane stability of spermatozoa, and therefore compromise their function. For this reason, we evaluated the plasma and mitochondria membranes of labeled-spermatozoa. Results summarized in Table 2 indicate that the presence of BRET-QD (0, 1, or 5 nm) had no effect on the proportion of sperm cells with intact membranes. In light of these data, it can be suggested that the incorporation of BRET-QD may not interfere with the normal progression of spermatozoa *in vivo* and interactions with its utero-oviductal environment, including the oocyte [9,31].

**Table 2:** Effect of BRET-QD on sperm viability

**Spermatozoa (10**^**8**^**)**	**Proportions of spermatozoa with intact:**			
**exposed to BRET-QD at:**	**N**	**Plasma membrane (%)**	**N**	**Mitochondrial membrane (%)**
0 nM	4	77.0 ± 3.3	3	95.8 ± 3.5
1 nM	4	78.8 ± 2.0	3	97.4 ± 1.7
5 nM	4	77.3 ± 2.3	3	97.0 ± 2.4
*P values (ANOVA-2)*		*P = 0.867*		*P = 0.906*

*N* Number of independent replicates. There were no significant differences between groups (p > 0.05). Data are means ± s.e.m.

**Table 3:** Fertilizing potential of BRET-QD labeled spermatozoa

**Groups**	**N**	**Proportion (%) of total oocytes analyzed as:**		**Total number of oocytes**
		**Fertilized**	**Unfertilized**	
Control^*^	4	63 ± 7	37 ± 7	179
Exposed	4	59 ± 9	41 ± 4	162

^*^Spermatozoa incubated without BRET-QD (1nM). *N* Number of independent replicates. There was no significant difference between groups (using the z-ratio for the significant difference between proportions*; P = 0.712*). Data are means ± s.e.m.

As the main function of the spermatozoa is to deliver the paternal materials to the oocyte, we further investigated the capability of BRET-QD labeled-spermatozoa to fertilize the oocyte. Based on the motility data above, we used spermatozoa (10^8^) labeled with 1 nM BRET-QD to fertilize *in vitro*-matured pig oocytes at a final concentration of 6x10^5^ sperm/ml. Table 3 shows comparable proportions of fertilized oocytes with unlabeled (control) and labeled (exposed) spermatozoa (63% ± 7% and 59% ± 9%, respectively; P > 0.05). These results indicated that the sperm labeling with sufficient amount of BRET-QD does not affect their fertilizing potential. Although the developmental performance of fertilized oocytes was not evaluated in our study, a recent report has demonstrated that exposure of oocytes to higher concentrations of CdSe core (125 nM) or CdSe/ZnS core-shell (500 nM) do not affect their developmental competence (fertilization, developmental and implantation rates and reduction of apoptosis and cell proliferation in blastocysts) [30].

## Conclusions

This study reports the possibility to label mammalian spermatozoa with bioluminescence resonance energy transfer CdSe/ZS quantum dot linked to the arginine-rich cell penetrating peptide R9. The results suggest that the self-illuminating BRET-QD can be employed for molecular imaging in mammalian spermatozoa without causing functional interference. Our results lay the ground work for implementing novel imaging techniques that can be utilized both for exploring important molecular characteristics of spermatozoa and for *in vivo* tracking of labeled-spermatozoa through a fluorescence endo-microscopy approach. The application of such imaging technology will allow a better understanding of sperm migration within the female genital tract.

## Methods

### Materials and reagents

A stock solution of CdSe/ZnS core-shell structure quantum dots (500 nM in Tris buffer) cross-linked to *Renilla* luciferase (BRET) and nona-arginine R9 peptide was purchased from Zymera Inc. (San Jose, CA, USA). The BRET-QD complex is a self-illuminating nanoparticle that emits light under incubation with coelenterazine (luciferase substrate; Zymera Inc.), and exhibits intense fluorescence with red-shifted emission (655 nm) following excitation. Boar semen was obtained from Prestage Farms (West Point, MS, USA) and oocytes from post-mortem gilt ovaries (South Quality Meats, Pontotoc, MS, USA).

### Sperm preparation and loading with BRET-QD

Freshly collected motile boar spermatozoa were selected as previously [32]. Spermatozoa (2 x 10^8^ sperm/ml) were incubated with 0, 1, or 5 nM BRET-QD at 37°C for 30 min. After three washes by centrifugation (1,000 g – 3 min) with PBS-PVP (1 mg/ml), supernatants containing excess QD were removed and 50 μl of each were kept for bioluminescence imaging. In parallel, sperm pellets were suspended with 50 μl PBS-PVP for experiments.

### Bioluminescence analysis

A total of 4 μg of coelenterazine was added to each cell suspensions and supernatants. All samples were imaged within around 5 min, but less than 10 min (photons/sec) using the IVIS 100 bioluminescence imager system (Caliper Life Sciences, Hopkinton, MA) with a 1 min acquisition time and without any filter.

### Detection of BRET-QD fluorescence emission within spermatozoa

Aliquots of spermatozoa incubated with 0, 1 or 5 nM BRET-QD were mounted onto microscope slides to evaluate their fluorescence emission. Samples were analyzed under a Laser Scanning Microscope system (LSM510, Carl Zeiss Micro Imaging GmbH, Jena, Germany) with a 488 nm excitation and 660/20 nm emission. The background fluorescence of samples without BRET-QD served as controls.

### BRET-QD localization in spermatozoa

Spermatozoa exposed to 0, 1, or 5 nM BRET-QD were suspended in phosphate-buffered 2.5% glutaraldehyde fixative solution. The standard protocol for sample preparation for transmission electron microscopy (TEM-JEOL) was performed without osmium tetroxide fixation and uranyl acetate staining [20]. Here, we excluded both steps in order to increase the background contrast and BRET-QD signal, and prepared samples of pure BRET-QD were placed on formvar-coated grids for TEM analysis. In parallel, aliquots of BRET-QD were placed on coated-slides to evaluate BRET-QD using Atomic Force Microscope (AFM) [18].

### Sperm motility and viability analyses

Immediately after incubation of spermatozoa and removal of the excess of BRET-QD, aliquots of spermatozoa were submitted to the motion analysis using a Computer Assisted Sperm Analyzer (CASA; IVOS v12; Hamilton Thorne Biosciences, Beverly, MA, USA). Motility characteristics of spermatozoa were determined using 20 μm4-chamber glass counting slides (Leja Products, Nieuw-Vennep, The Netherlands).

Additional aliquots of labeled or non-labeled spermatozoa were used for viability analyses after staining of cells for either plasma (Propidium Iodide; 10 μg/ml; Sigma-Aldrich Co., Saint Louis, MO, USA) or mitochondrial (JC-1; Cayman Chemical Co., Ann Arbor, MI, USA) membrane integrities. The proportions of viable spermatozoa were evaluated with a flow cytometer (Becton Dickinson FACSCalibur) set for 10,000 total events per analysis.

### Fertilizing potential of spermatozoa

Ovaries were collected from post-mortem gilts and oocytes were selected and matured *in vitro* according to Feugang et al. [32]. Matured oocytes were fertilized at a final concentration of 6 × 10^5^ spermatozoa/ml with spermatozoa (10^8^) pre-exposed to 0 or 1 nM BRET-QD diluted in PBS-PVP (1 mg/ml). After 18 h co-incubation, the proportions of fertilized and non-fertilized oocytes were evaluated as previously reported [33].

### Data analyses

Each experiment was repeated at least three times. Fertilization data were analyzed using the z-ratio, to evaluate the significance of the difference between proportions in the control and exposed (1nM BRET-QD) groups. Bioluminescence, motility, viability and velocity data were analyzed using the two-way ANOVA that considered both replicates (N) and groups. Pairwise comparisons were performed using the Fisher’s LSD test. Data are expressed as mean ± s.e.m., unless otherwise indicated. The threshold of significance was set at *P ≤ 0.05* and tendency at *P < 0.1*.

## Abbreviations

PBS: Phosphate-buffered solution; PVP: Polyvinylpyrrolidone.

## Competing interests

The authors declare that they have no competing interests.

## Authors’ contributions

JMF conceived the study, designed, performed most of the experiments, and drafted the manuscript; RCY and JMG contributed to the experimental design and participated to the bioluminescence imaging; WAM carried out the TEM analysis and helped for confocal microscope imaging; ASF performed the sperm motility analysis; PLR and STW participated to the experimental design and provided guidance; All the authors have discussed the results and reviewed the manuscript. All authors read and approved the final manuscript.
